# A low-cost homogenizing device for in-field and remote DNA and RNA extraction

**DOI:** 10.1016/j.ohx.2024.e00592

**Published:** 2024-10-09

**Authors:** Christelle Schang, Le Zhang, Baiqian Shi, Monica Nolan, Rachael Poon, David T. McCarthy

**Affiliations:** a BoSL Water Monitoring and Control, Department of Civil Engineering, Monash University, Victoria 3800, Australia; b Department of Health Victoria, Melbourne, Victoria 3000, Australia; c School of Civil and Environmental Engineering, QUT, Brisbane, QLD 4001, Australia; d School of Environmental Sciences, Ontario Agricultural College, University of Guelph, Canada

**Keywords:** 3D printing, Bead beater, Remote processing, Wastewater-based epidemiology, WBE, Portable, Environmental sampling

## Abstract

Environmental monitoring of microorganisms is critical for the protection and enhancement of human and ecosystem health. Even though these molecular methods have overtaken traditional culture-based methods and become more accessible, these techniques still require expensive equipment and dedicated facilities to process samples which in the context of a global pandemic, remote sampling areas or low-income countries can be extremely challenging. Sample preparation and sample homogenisation are critical steps for molecular-based techniques, especially for the extraction of DNA and RNA. This study developed a low-cost, open-source, freely available 3D printed homogenizer for the processing of DNA and RNA extraction. The BoSL Beater 3D is a portable device that allows researcher to perform bead-beating steps commonly required for environmental sample extraction protocols in the field and without access to main’s power. The BoSL Beater 3D was tested on filtered wastewater samples and passive samplers exposed to wastewater over a 24-hour period and showed similar or better performance to the traditional laboratory bead beater for both the extraction of DNA and RNA. The cost of this 3D homogeniser is roughly $18 AUD ($296 AUD with the jigsaw, which is roughly 57 times cheaper than a traditional bead beater) and has the added usability of being portable and easily adaptable to any type of jigsaw. In combination to newly developed field extraction kits as well as portable PCR machines, this 3D homogeniser could provide the tool necessary to enable access to molecular testing in remote setting as well as developing countries, which may not have access to fully equipped laboratories, but also allow for timely reporting. In addition, the BoSL Beater 3D, in combination with field extraction kit, can allow more flexibility to researchers while sampling, shipping, and processing DNA and RNA samples, whilst maintaining quality of these samples.

## Graphical abstract

**Figure:**
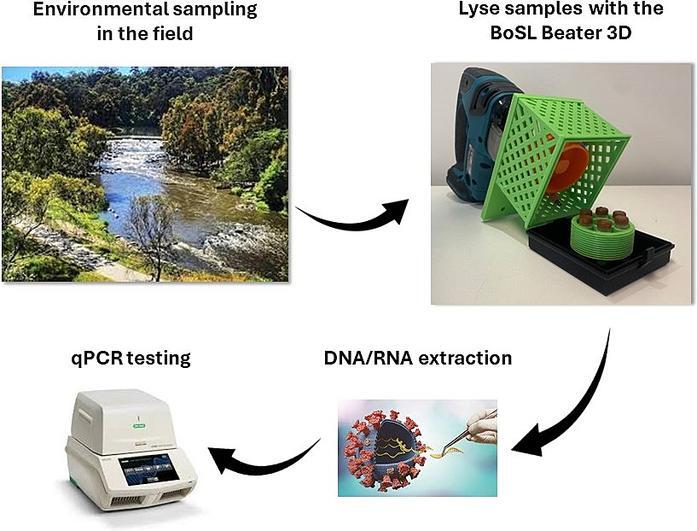

## 1 Introduction

Specifications table

**Table:** 

Hardware name	**BoSL Beater 3D**
Subject area	• Engineering and materials science • Biological sciences (e.g., microbiology and biochemistry) • Environmental, planetary and agricultural sciences
Hardware type	• Biological sample handling and preparation • Mechanical engineering and materials science
Closest commercial analogue	• MPBio FastPrep-24™ 5G bead beating grinder and lysis system (USA, approximately $16,900AUD) • MPBio SuperFastPrep-2™, portable bead beating grinder and lysis system (USA, approximately $8,690 AUD) • Qiagen PowerLyzer 24Homogenizer (Germany, $6,400–8,600 AUD), • Qiagen TissueLyzer II (Germany, $5,700–8,600 AUD), • Benchmark Scientific BeadBlaster Microtube Homogenizer ($13,400–21,300 AUD), • Qiagen Vortex-Genie + Adapter ($1,100 AUD) • PortaLyser, ([1], approximately $200USD)
Open-Source license	Creative Commons Attribution 4.0 International (CC BY 4.0)
Cost of hardware	$296 AUD including the Makita jigsaw used in this study (design can easily be adapted to other jigsaws): $18 AUD case + holder without jigsaw
Source file repository	https://doi.org/10.17632/x2gpn7sjyt.1

## 2 Hardware in context

Environmental monitoring of microorganisms is critical for the protection and enhancement of human and ecosystem health. Wastewater surveillance, also known as wastewater based epidemiology (WBE), has played a critical role in managing the COVID-19 pandemic by tracking disease dynamics at various scales around the world [2], [3], [4], [5]. Meanwhile, monitoring of environmental Antimicrobial Resistance (AMR) is greatly improving our understanding of the dissemination routes of resistant microorganisms outside of clinical and veterinary settings [6].

Molecular-based environmental microbiology has overtaken traditional culture methods due to its less invasive nature and reduced turnaround times. Polymerase chain reaction (PCR)-based molecular methods are sensitive and quantitative, allowing us to study the environmental dynamics of bacterial, protozoan and viral pathogens, including both new and emerging strains [7]. New portable PCR technologies, such as the Biomeme Franklin thermocycler (Biomeme, USA) [8], [9] or the Liberty16 (Ubiquitome, USA [10]) are rapidly changing our ability to monitor disease, enabling applications in highly remote regions, low-income countries and informal settlements of the globe. Unfortunately, portable equipment required to pre-treat and prepare environmental samples for PCR remains limited in accessibility.

Nucleic extraction of environmental samples is a complex process and is often confined to laboratory facilities that have expensive and difficult to procure equipment that require expert use [11], [12], [13], often beginning with a critical homogenization step to lyse cells, grind, agitate and mix samples. Even in lab settings, we often neglect the importance of selecting an appropriate mechanical homogenizer, the growing body of evidence indicating that homogenization is a crucial first step in the experimental workflow [12] and that mechanical homogenization has a tremendous impact on the experimental outcome particularly with regards to reproducibility [14], [15]. Apart from simple vortexers, commercial options exist to homogenize samples (such as MPBio FastPrep-24™, Qiagen PowerLyser 24 Homogenizer or TissueLyser II and Benchmark BeadBlaster Microtube Homogenizer) but these options are expensive (for example: FastPrep-24™ 5G, $16000 AUD) and not portable (weight > 15 kg). Beyond the comfort of the laboratory, the development of field-portable homogenizers has received little attention, despite the growing number of newly developed portable PCR tools that should require these devices as a precursor.

To the authors’ knowledge, the TerraLyser™ (Zymo Research, USA) was the only portable homogenizer that was commercialized and did not require mains-power, but it was discontinued due to mechanical issues and battery usage and could only process one sample at a time. More recently, a new low cost vortex instrument was researched and has showed equivalent DNA quality and yield as those processed with a traditional laboratory vortex [1]. However, this device had several drawbacks: (1) its homogenization step takes 20 min making it difficult to process a larger number of samples and (2) the homogenization step is based on simple vortexing, which in comparison to bead-beating has a reduced efficiency to lyse cells and agitate samples [13]. Indeed, vortexing alone only provides one, circular motion continuously and usually at a constant speed, so as sample and beads are set in motion, the incidence of direct impact of grinding bead to sample is relatively small, whilst bead-beating, such as the FastPrep™ 24 instruments, combine cascade impaction, mechanical shearing and hydrodynamic/vortex shearing providing a more effective impact in a shorter period of time [13].

To our knowledge, there are no publications that report on the development and testing of a portable, battery-powered, bead beating device that can appropriately extract DNA and RNA from complex environmental samples. As such, this paper presents an open hardware, open-source bead- beater that is small, cheap, portable and quick to construct whilst relying on easily available equipment and consumables and a commonly used power tool. This study aimed to provide proof of concept of a low-cost bead beater for in-situ testing by comparing its performances to a commercially available lab-quality bead beater.

## 3 Hardware description

The BoSL Beater 3D is a 3D printed sample homogenizer designed for the processing of environmental samples which can be inserted in 2 mL extraction tubes. The samples can be soil, faeces, passive materials (such as 47 mm membranes, cotton buds, cotton gauze etc.), water samples filtered through 47 mm membranes. We believe that this 3D printed sample homogenizer offers many advantages over traditional laboratory homogenizers:

• low cost (the initial cost of the jigsaw and 3D attachment is on average 60 times cheaper than a traditional laboratory homogenizer, whilst the 3D attachment itself although less durable is 1500 times cheaper),
• easily constructed from readily available materials,
• added usability of being portable, no need for designated laboratory space,
• adaptable to any commercially available jigsaw,
• more accessible to low-income countries,
• more accessible to remote areas,
• allows easier transport of samples across country borders once DNA/RNA has been isolated.

**Fig. 1:**
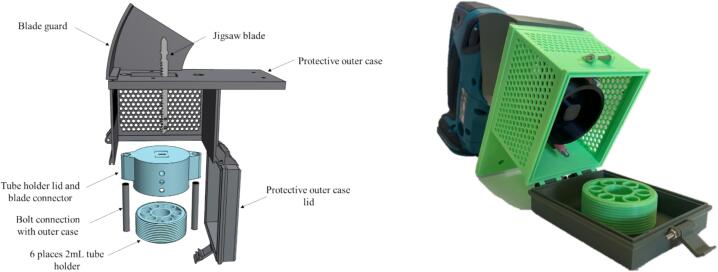
Left: The BoSL Beater 3D bead beater design (Shapr3D version 5.471.5829.0), Right: photograph of the bead beater installed on the Makita Jigsaw (Matika MODEL DJV180, Japan). This model was design to fit standard 2 mL extraction tubes. Note that the device has a protective guard to ensure that the jig blade is not reachable by hands.

The final design of the BoSL Beater 3D homogeniser (Fig. 1) is comprised of: (1) a 3D-printed, six-place tube holder equipped with a lid, (2) a 3D-printed outer protective box and (3) a protective guard to prevent access to the jigsaw blade when operating the instrument. A protective case was designed with mesh walls to ensure the operators could check that the instrument was performing as expected and could stop the process immediately if parts were to get loose or not moving as expected. An alternative protective case with 100 % infilled walls was also designed and could be printed with transparent filament if necessary (3D renderings of the different options are available in Fig. S1 in Supplementary material and design files are listed in Table 1). All design files are available and free to download from the online depository as listed in the design file summary table (Table 1). The 3D printed device can process six samples at a time and can be attached to any commercially available jigsaw which enables the homogenizing function. The outer case and the container lid ensure the vertical movement of the jigsaw and secure the tubes in place. Using high-speed video recording and the Makita LXT jigsaw characteristics of 2600 movements per min and a movement of 2.6 cm for each full movement of the blade, the speed of the 3D homogenizer was estimated at 1.13 m/s. This speed is about 3.9 times slower than the MPBio FastPrep™ 24 5G laboratory bead beater setting used in this study (hereby FastPrep24) (Table 2).

**Table 1:** Design file summary.

**Design file name**	**File type**	**Open-source license**	**Location of the file**
BoSL_Beater_3D_final_design_all_parts.shapr	*shapr*	CC BY 4.0	https://doi.org/10.17632/x2gpn7sjyt.2
BoSL_Beater_3D_final_design_all_parts.x_t	Parasolid Model part file	CC BY 4.0	https://doi.org/10.17632/x2gpn7sjyt.2
BoSL_Beater_3D_final_case_mesh.stl	STL	CC BY 4.0	https://doi.org/10.17632/x2gpn7sjyt.2
BoSL_Beater_3D_final_case_solid.stl	STL	CC BY 4.0	https://doi.org/10.17632/x2gpn7sjyt.2
BoSL_Beater_3D_final_case_lid + latch.stl	STL	CC BY 4.0	https://doi.org/10.17632/x2gpn7sjyt.2
BoSL_Beater_3D_blade_guard.stl	STL	CC BY 4.0	https://doi.org/10.17632/x2gpn7sjyt.2
BoSL_Beater_3D_lid and holder.stl	STL	CC BY 4.0	https://doi.org/10.17632/x2gpn7sjyt.2
BoSL_Beater_3D_dataset.xlsx	Microsoft Excel	CC BY 4.0	https://doi.org/10.17632/x2gpn7sjyt.2

BoSL_Beater_3D_final_design_all_parts.shapr. An original design file used to create a 3D model of the BoSL Beater 3D using Shapr3D version 5.471.5829.0. The choice of this software was made due to the additional functionality to draw better threads and incorporate the bolts in the design. This file contains both the honeycomb case and the solid wall case.

BoSL_Beater_3D_final_design_all_parts.x_t. An original Parasolid model part file which can be used with different CAD programs to visualise and modify the 3D model of the BoSL Beater 3D.

BoSL_Beater_3D_final_case_mesh.stl: Standard Tessellation Language (STL) ready to print component file for for the honeycomb wall version of the BoSL Beater 3D protective case.

BoSL_Beater_3D_final_case_solid.stl: Standard Tessellation Language (STL) ready to print component file for the solid wall version of the BoSL Beater 3D protective case.

BoSL_Beater_3D_final_case_lid + latchlatch.stl: Standard Tessellation Language (STL) ready to print component file for the BoSL Beater 3D case cover and closing latch.

BoSL_Beater_3D_blade_guard.stl: Standard Tessellation Language (STL) ready to print component file for the BoSL Beater 3D blade guard component.

BoSL_Beater_3D_lid and holder.stl. Standard Tessellation Language (STL) ready to print component file for the tube holder and bead beater lid of the BoSL Beater 3D.

BoSL_Beater_3D_dataset.xlsx. A spreadsheet containing validation data.

**Table 2:** Bill of materials (in AUD).

**Designator**	**Component**	**Number**	**Cost per unit −currency**	**Total cost −** **currency**	**Source of materials**	**Material type**
*Protective case with lid and closing latch*	*1.75 mm 3D* *printing filament*	*172 g*	*$0.04 AUD*	*$6.84 AUD*	*https://www.b3d.com.au/DispProd.asp?ProdID=PLA175GreenLt1*	*PLA*
*Blade guard*	*1.75 mm 3D* *printing filament*	*44.8 g*	*$0.04 AUD*	*$1.79 AUD*	*https://www.b3d.com.au/DispProd.asp?ProdID=PLA175GreenLt1*	*PLA*
*Tube holder lid, 6 places tube holder,* *Outer sleeves for M6 bolt*	*1.75 mm 3D* *printing filament*	*138.2 g*	*$0.04 AUD*	*$5.53 AUD*	*https://www.b3d.com.au/DispProd.asp?ProdID=PLA175GreenLt1*	*PLA*
*Outer protective case attachment to jigsaw* *(Alternative screws Counter sunk head (C.S.H) screw M4, length 8 mm included with the jigsaw)*	*Machine Screw, M4, 12 mm, Stainless Steel A2, Pan Head Pozidriv*	*4*	*$0.05 AUD*	*$0.20 AUD*	*https://au.element14.com/tr-fastenings/m412-pra2mcs100/screw-pozi-pan-s-s-a2-m4x12-pk100/dp/1420040*	*Stainless steel A2*
*Protective case to lid connection*	*Bolt M4, length* *35 mm with M4 nuts*	*2*	*$0.33 AUD*	*$0.66 AUD*	*https://www.pinnacle.net.au/product/round-bolts-nuts-m4-x-35* *mm-black-ruspert/*	*Stainless steel*
*Closing latch to protective case*	*Bolt M3, length 32 mm with M3 nuts*	*2*	*$0.33*	*$0.66AUD*	*https://www.jaycar.com.au/m3-x-32* *mm-steel-screws-pack-of-25/p/HP0418*	*Stainless steel*
*Protective outer case to jigsaw base connection*	*Bolt M6, length 75 mm*	*2*	*$0.26 AUD*	*$0.52 AUD*	*https://au.element14.com/tr-fastenings/m3-16-prstmc-z100/screw-pozi-pan-steel-bzp-m3x16/dp/1420393*	*Stainless steel*
*Protective outer case to jigsaw base connection*	*Self-locking nut M6*	*2*	*$0.28 AUD*	*$0.56 AUD*	*https://au.element14.com/tr-fastenings/m6-n5a4-s50/nyloc-nut-s-s-a4-m6-pk50/dp/1420450*	*Stainless steel*
*Unit to jigsaw blade connection*	*Bolt M3, length 16 mm*	*2*	*$0.03 AUD*	*$0.06 AUD*	*https://au.element14.com/tr-fastenings/m3-16-prstmc-z100/screw-pozi-pan-steel-bzp-m3x16/dp/1420393*	*Stainless steel*
*OPTIONAL (design can be adapted to any jigsaw available on the market by adjusting the attachment of the outer case)*						
*Jigsaw*	*Makita Jigsaw*	1	*$279.00 AUD*	*$279.00 AUD*	*https://www.amazon.com.au/makita-D-Handle-Jigsaw-Multicolour-SMALL/dp/B00IILRQBY?ref_=ast_sto_dp&th* *= 1&psc = 1*	*Battery operated tool*
*Jigsaw blade*	*Blade (product code: D-72718)*	1	*$1.44 AUD*	*$1.44 AUD*	*https://sydneytools.com.au/product/makita-d72718-5pack-100* *mm-9tpi-jigsaw-blade-for-wood*	*High carbon steel*

## 4 Design files summary

The 3D models and *.stl files for each part of the BoSL Beater 3D are listed in Table 1.

## 5 Bill of materials

The cost of the different components and materials for the BoSL beater 3D are summarised in Table 2. All costs are reported in AUD.

**Table 3:** Summary of bead beating conditions tested on different type of samples. The lab bead beater used was the MP Bio FastPrep-24 5G. “-“ means that the condition was not tested for this type of sample. “×” indicates that the bead beating condition was tested for a specific type of sample. The beat beating condition are described as duration of the bead beating step, speed of beat beating step, type of bead beater used if used.

	**Sample type**	
**Bead beating condition**	**Passive sampler CN membrane (24hr exposure)**	**20mL wastewater on CN membrane**
45 sec, 6.5 m/s, lab bead beater	×	×
60 sec, 6.5 m/s, lab bead beater	−	×
2×60 sec, 6.5 m/s, lab bead beater	×	−
10 sec, 6.5 m/s, lab bead beater	−	×
45 sec, 3D bead beater	−	×
60 sec, 3D bead beater	−	×
2×60 sec, 3D bead beater	×	−
10 sec, 3D bead beater	−	×
10 manual shakes (no bead beater)	−	×

## 6 Build instructions

*3D design.* The device was designed using Shapr3D (version 5.692.7363.0, Shapr3D Zrt., Hungary) to fit the dimension of commonly used 2 mL garnet bead tubes and to be adapted on a Makita LXT Jigsaw (Makita, Japan), but simple changes to the 3D print design files could be made to adapt to other brands of jigsaws.

*3D printing.* The design file (BoSL_Beater_3D_full_design.x_t) is exported into three Standard Tessellation Language files (BoSL_Beater_3D_case.stl, BoSL_Beater_3D_Cover_Case.stl and BoSL_Beater_3D_lid and holder.stl) which are then loaded into the printer software (FlashPrint 4.0.0, FlashForge). The filament used is made of Polylactic acid (PLA) plastic, 1.75 mm in diameter. Other materials, such as polyethylene terephthalate glycol (PET-G) and Acrylonitrile butadiene styrene (ABS), have been successfully used but were not tested as part of this study. We use a FlashForge CreatorPro with FlashPrint 4.0.0 software and the printing parameters for the PLA material were as follow:

- **Protective outer case and cover case**: fine slice profile, 20 % fill density, line pattern, combining every 2 layers, first layer height set to 0.2 mm, 4 perimeters shells, 6 top solid layers, 6 bottom solid layers, layer height 0.12 mm, print speed of 50 mm/s and travel speed of 70 mm/s, extruder temperature 225 °C, bed temperature 60 °C, no wall or raft were used;
- **Screw top lid and blade connector**: standard slice profile, 30 % fill density, line pattern, combining every 2 layers, first layer height set to 0.2 mm, 3 perimeters shells, 4 top solid layers, 3 bottom solid layers, layer height 0.18 mm, print speed of 60 mm/s and travel speed of 80 mm/s, extruder temperature 220 °C, bed temperature 60 °C, no wall or raft were used;
- **Screw tube holder base**: fine slice profile, 100 % fill density, line pattern, combining every 2 layers, first layer height set to 0.2 mm, 4 perimeters shells, 6 top solid layers, 6 bottom solid layers, layer height 0.12 mm, print speed of 50 mm/s and travel speed of 70 mm/s, extruder temperature 225 °C, bed temperature 60 °C, no wall or raft were used.

Using these printing conditions, the total printing time for the protective case, the screw top lid and the screw tube holder base are 38hrs, 4hrs and 10hrs respectively. After printing, all parts are cleaned from excess printing material using a sharp edge tool (e.g., scissors or cutters).

**Fig. 2:**
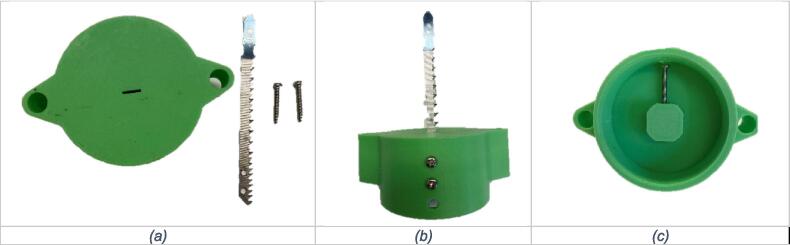
Assembly of the BoSL Beater 3D homogenizer blade to tube holder lid connection: (a) 3D printed lid, jigsaw blade with two assembly holes and the two M3 bolts (b) jigsaw blade in position in the 3D printed lid and two bolts in position ready to be secured (c) details of bottom screw location and how to insert it.
2) Insert the two stainless steel M6 bolts through the two larger holes (Fig. 3b).

*Assembly.* The assembly of the device can be completed by following these steps:

1) Assemble the BoSL Beater 3D tube holder lid by inserting the jigsaw blade into blade slot and secure using the two M3 x 16 mm bolt (Fig. 2).

**Fig. 3:**
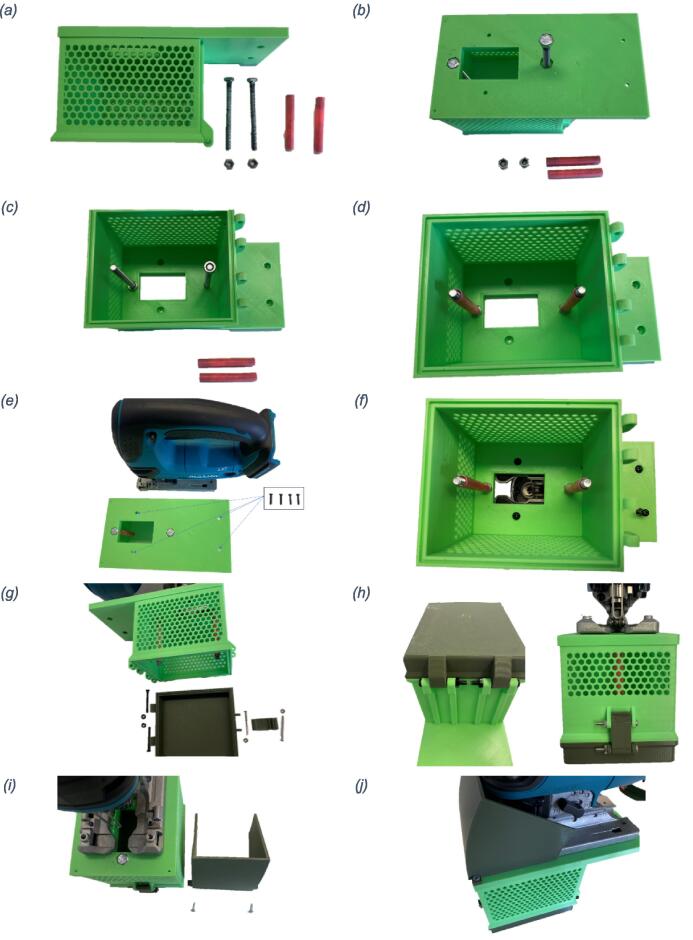
Assembly of the BoSL Beater 3D homogenizer protective case (a) components needed for this step, (b) insertion of the two M6 bolts in the printer case, (c) Secure bolts with self-locking nuts inside the case, (d) screw the 3D printed bolt sleeves onto the M6 bolts (e) line up jigsaw and outer case to insert the 4 machine screws M4 (f) insert the four M4 screws to attach outer case to jigsaw (g) outer case lid with latch, bolts and nuts (h) lid to outer case connection details (i) components of the blade guard (j) blade guard in place.
3) Secure the two stainless steel M6 bolts using the two M6 self-locking nuts and place the two 3D-printed plastic sleeves on the bolts (Fig. 3c and d).
4) Attach protective case to jigsaw using the four M4 machine screws through the four smaller holes previously lined up in position with the jigsaw (Fig. 3e and f).
5) Attach protective case’s lid using two M4 x 35 mm bolt and M4 self-locking nuts.
6) Attach latch to the protective case’s lid using a M3 x 35 mm bolt and M3 nut. Attach the second M3 x 35 mm bolt onto the protective case and secure using M3 nut (Fig. 3g and h).
7) Attach blade guard to jigsaw using two 4 g x 12 mm screws (Fig. 3i and j). We recommend performing this step after having inserted the BoSL Beater 3D homogenizer tube holder component (displayed in Fig. 2).

## 7 Operation instructions

1) Ensure the jigsaw battery is not connected to the power tool;
2) Open the protective casing and unscrew tube holder;
3) Place the sample tubes into the holder and ensure it is balanced;
4) Screw the holder back to the holder lid tightly;
5) Close the protective case;
6) Connect the jigsaw battery;
7) Start bead beating process using the jigsaw for the desired duration;
8) To remove the tubes, repeat Steps 1 and 2 and reverse Step 3.

For safety, ensure all parts are in good condition before use and that the jigsaw is not powered (i.e., the battery is disconnected when inserting and removing the tubes from the tube holder). The jigsaw being a loud handheld power tool, wearing earplugs/earmuffs and protective safety googles while the using the device is recommended.

## 8 Validation and characterization

The operation and efficiency of the BoSL Beater 3D was characterized and validated using a series of four laboratory tests using wastewater samples and comparing the results obtained to those obtained using a traditional laboratory bead beater. A total of 57 x 20 mL of wastewater, collected from a local wastewater treatment plant in Melbourne, Australia, were filtered on 0.45 µm cellulose nitrate electronegative membranes (hereafter CN membranes, Sartorius, Germany). All samples were filtered on the same day. An additional 20 CN membranes were submerged into 1L of wastewater and placed into an automated tumbler and continuously mixed at 100 rpm to simulate the adsorption process of passive samplers for a period of 24 hrs (for more information about passive sampling, see [16]). All CN membranes were stored at −80 °C until extraction was performed.

During each extraction, each CN membrane was placed directly into a 2 mL tube containing 0.7 mm garnet beads and after homogenisation they were all extracted using the MagMax Microbiome Ultra Nucleic Acid Purification kit (Applied Biosystems, USA) via the Kingfisher Apex instrument (Thermo Fisher Scientific, USA). Seven different homogenisation methods were tested using triplicate samples of filtered wastewater (or five replicates in the case of the passive samplers) (Table 3); importantly, we compared the BoSL-Beater 3D, a 3D printed bead beater, to a commercially available “lab” FastPrep-24™ 5G bead beating grinder and lysis system (MPBio, USA). For the remainder of the extraction protocol, manufacturer’s instructions were followed. To assess the extraction performances of each homogenisation method, a sample processing control sequence (SPC) taken from chum salmon, *Oncoryhynchus keta,* was added into each tube at the start of the extraction at a final concentration of 0.2 µg/mL as described in the US EPA Method 1696 [17]. Additionally, 10 µL of MS2 phage were added directly into each tube as described in Schang et al. [16]. For each homogenisation condition, a method extraction blank (MEB) made of 20 mL of nuclease free water (Qiagen, Germany) filtered on the same CN membrane as the wastewater samples or a dry CN membrane was processed in the case of the passive samplers.

The quantity and quality of all extracted material was assessed using a Denovix DS-11 FX spectrophotometer/fluorometer instrument (Denovix, USA). The purity of the samples was analysed using the A_260_/A_280_ ratio which is a measure of DNA and RNA purity and the A_260_/A_230_ ratio which indicates the presence of unwanted organic compounds and chaotropic salt.

All extracted material was tested for SARS-CoV-2 N and ORF-1ab genes, an internal control (IC) MS2 phages and pepper mild mottle virus (PMMoV) using a modified Perkin Elmer SARS-CoV-2 RT-qPCR kit (Perkin Elmer, USA). We tested for *Oncoryhynchus keta* (Sketa 22) following the US EPA Method 1696 [17] to assess the full extraction efficiency of each homogenisation method. All samples were run in duplicate using Bio-Rad CFX 96 instrument (Bio-Rad, USA) and Cq values were obtained and analysed using the Bio-Rad CFX Maestro software (Bio-Rad, USA, software version 2.3). The details about the primers and probes, mastermix and RT-qPCR conditions are provided in Supplementary material (Real-Time quantitative PCR section). The results from each PCR runs were standardised based on a high number of standard curve experiments (see [16]); as per this reference, we adjusted the RFU threshold of each channel: 500 RFU were used for the N gene, ORF-1ab gene and IAC (MS2 phage), 400 RFU were used for PMMoV and 1300 RFU were used for Sketa22.

Statistical analysis was performed using GraphPad Prism (version 9.0.1 (1 5 1), GraphPad Software, LLC) and Microsoft Excel (Version 2407, Microsoft 365 MSO) to assess the impact of the homogenisation methods, durations of the homogenisation and sample types on the DNA and RNA quality and the Cq values for the different organisms of concern. The data set had to be fractioned as the experimental design was not full-factorial (Table S3 −Supplementary material). The data was checked for normality using Shapiro-Wilks test and the assumption of homoscedasticity was checked using Levene’s test. The data showed less than 2 % of outliers based on the ROUT test with a False Discovery Rate (Q) of 0.1 %. No data was systematically discarded using outlier rejection rules. As the data was found not to follow a normal distribution, Kruskal-Wallis’s test was used to assess the significance of the homogenisation steps on each of the factors tested. When significant difference was observed, the Dunn’s test was used to assess which pair of means were different.

**Fig. 4:**
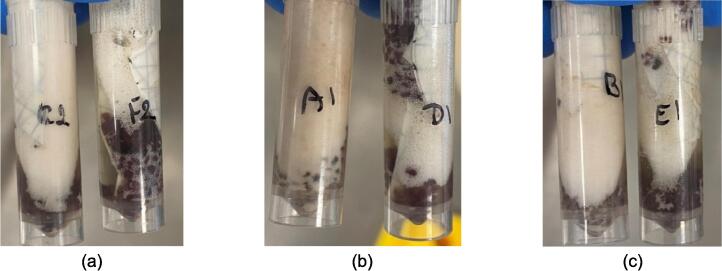
Filtered 20 mL wastewater lysates after (a) 10 sec, (b) 45 sec and (c) 60 sec of bead beating using the FastPrep-24 left and the BoSL Beater 3D right of each picture.

*Mechanical shearing assessment:* Visually, the BoSL Beater 3D seems to cause less shearing of the garnet bead matrices contained into each of the 2 mL tubes as well as less elution buffer foaming especially for duration of 45 to 60 s and less resuspension of the material attached onto the electronegative membranes for the 20 mL wastewater filtered samples (Fig. 4).

**Fig. 5:**
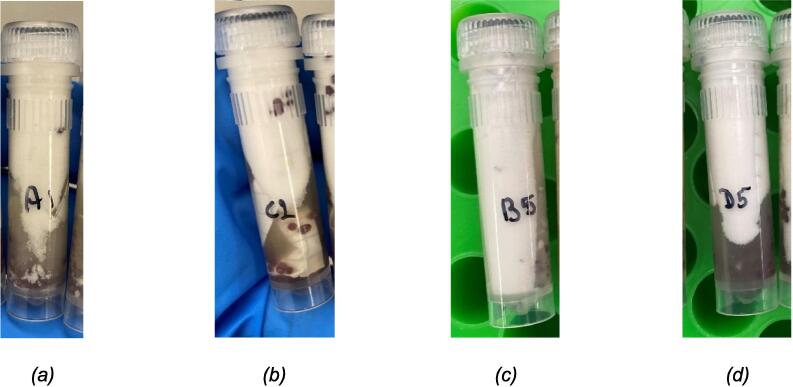
Passive sampler lysates after (a) 45 sec with FastPrep-24, (b) 45sec with BoSL Beater 3D and (c) 2×60sec with FastPrep24 5G (d) 2×60sec with BoSL Beater 3D.

Similarly, the BoSL Beater 3D seems to cause less shearing of the garnet bead matrices contained into each of the 2 mL tubes and less lysis buffer foaming especially for both 45 sec and 2 x 60 sec of homogenization in the case of passive sampler membranes (Fig. 5). This combined with the fact that the passive samplers in this experiment did not have a lot of foreign material attached to them mean that there was probably less resistance to break open the cell and therefore a “gentler” cascade impaction process in the case of the BoSL Beater 3D.

*DNA and RNA quality and quantification:* More detailed summary statistics and discussions of the DNA and RNA quality measurements for both the wastewater samples and the passive samples are provided in Supplementary material (Table S4 and S5). Good-quality DNA will have an A_260_/A_280_ purity ratio of 1.7–2.0 and a A_260_/A_230_ ratio greater than 1.5 and ideally within the range of 2.0–2.2 [18]. Overall, the use of the BoSL Beater 3D resulted in samples of higher DNA and RNA purity than those obtained from the FastPrep-24 for the wastewater samples based on the A_260_/A_280_ ratios and the A_260_/A_230_ ratios. The only time the FastPrep-24 outperformed the BoSL Beater 3D was for the DNA purity ratios A_260_/A_280_ for the passive samples (average A_260_/A_280_ = 1.91 vs A_260_/A_280_ = 1.57 for FastPrep-24 and BoSL Beater 3D respectively). The duration of the bead beating was the most significant factor impacting purity of the DNA and RNA extracts with longer bead beating resulting in poorer quality. The only exception was for the A_260_/A_230_ ratio for the passive samplers processed using the BoSL Beater 3D for 2 x 60 sec which were the only passive sampler combination to be within the range of 2–2.2, all other ratio were below 2 for both DNA and RNA, showing the potential presence of some contaminants such as residual guanidine from the MagMax Microbiome extraction kit [18].

**Fig. 6:**
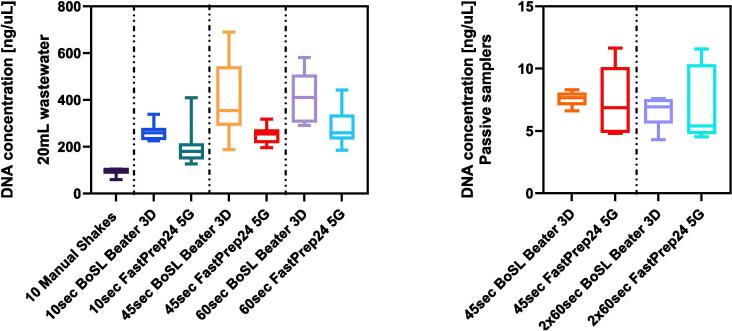
DNA yields for 20 mL wastewater samples (left) and 0.45 µm passive samplers' CN membranes (right) obtained from different homogenisation method (duration and homogenisation instrument). The boxplots display the pooled data from all four tests conducted.

*DNA and RNA quantification:* DNA and RNA concentrations were measured for each of the samples tested using a spectrophotometer/fluorometer (DeNovix, model DS-11FX, USA). For the filtered wastewater samples, we found that DNA yield was statistically lower for the manual shaking as compared to either of the mechanical methods (p < 0.05), thus showing the importance of beat-beating as a method of homogenisation during extraction. The wastewater samples that were manually shaken resulted in a DNA yield 2.3 to 4.8 times lower than the samples processed using the two mechanical methods (Fig. 6 and Table S4). Importantly, the DNA yield of the wastewater samples extracted using the BoSL Beater 3D was on average 1.46 times higher than the yield from the FastPrep-24 for the same homogenization duration (Fig. 3). Statistical analyses reinforced this observation, showing that the BoSL Beater 3D statistically outperformed the laboratory device under all conditions for 20 mL wastewater samples. For the passive samplers, DNA yields from the two mechanical devices were not statistically different (*Kruskal-Wallis*, p > 0.05), however a much smaller standard deviation was observed for the BoSL Beater 3D (Fig. 6 – right) suggesting a superior repeatability as compared to the lab device. Our analyses also showed that homogenization duration was also seen to be a significant factor (p < 0.001) and both the FastPrep-24 and the BoSL Beater 3D had the highest DNA yield for a 60 sec homogenisation step and the lowest yield for a 10 sec homogenisation step. There was no significant difference between 45 and 60 sec of homogenisation for either the FastPrep-24 (p = 0.4) and the BoSL Beater 3D (p = 0.55) (Fig. 6).

**Fig. 7:**
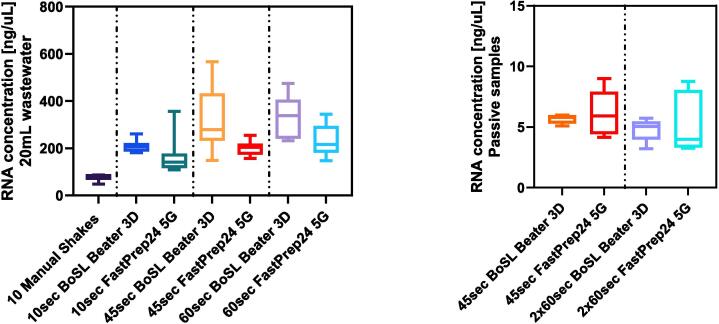
RNA concentrations for 20 mL wastewater samples (left) and 0.45 µm passive samplers' CN membranes (right) obtained from different homogenisation method (duration, and homogenisation instrument). The boxplots display the pooled data from all four tests conducted.

Overall, similar observations could be made for the RNA yields of the wastewater and passive samplers’ samples (Fig. 7). On average the RNA yield of the wastewater samples processed using the BoSL-Beater 3D for the homogenisation step were 1.44 higher than those processed using the FastPrep-24 instrument (Table S5 - Supplementary information), whilst there was no significant difference between the two methods for the passive samplers (*Kruskal Wallis test*, p > 0.05)*.* The results showed once again the importance of beat-beating as a method of homogenisation, as the RNA yields were all significantly higher than the samples manually shaken (p < 0.05) except for the 10 sec homogenisation using the FastPrep-24. A 60 sec homogenisation step, showed to results in the highest average concentration for both homogenisers, however the difference between 45 sec and 60 sec was not significantly different (p > 0.3).

In conclusion, whilst DNA and RNA concentration were slightly higher for both homogeniser with a 60 sec step, both the DNA and RNA quality ratios were lower than when homogenising the sample for 45 sec. And therefore a 45 sec homogenisation step would be recommended for wastewater samples and passive samplers. Although purity ratios and spectral profile are important indicators of sample quality, the best indicator of DNA and RNA quality is functionality in the downstream applications of interest, in this case quantification by PCR.

**Fig. 8:**
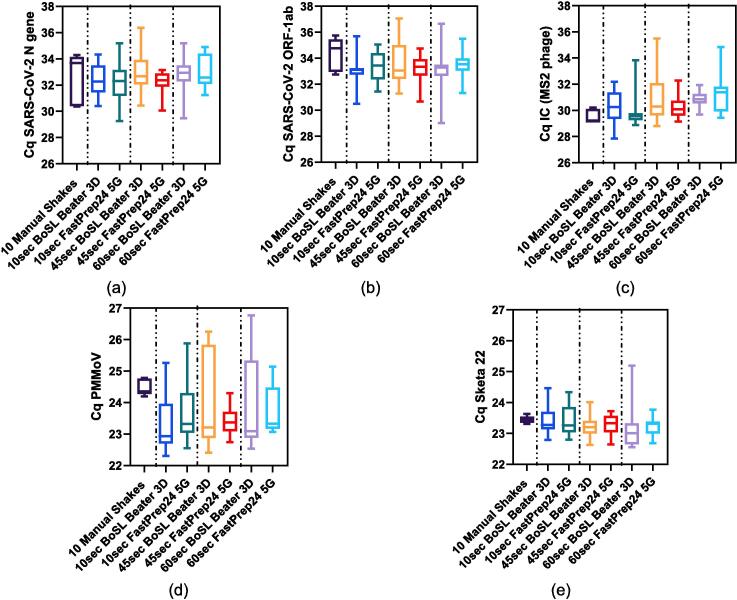
Combined Cq values (n = 18) for **20 mL wastewater samples** filtered on 0.45 µm CN membrane for (a) SARS-CoV-2 N gene, (b) SARS-CoV-2 ORF-1ab gene, (c) Internal control – MS2 phage, (d) PMMoV and (e) Sketa22. Each boxplot represents the combined data of three trial test for a given homogenisation method.

*Extraction efficiency and qPCR for filtered wastewater samples:* The combined data from all experiments is presented in boxplots in Fig. 8 and summary statistics are presented in Table S6 of the Supplementary material. With the exception of MS2, the manual shaking method of homogenisation resulted in the lowest average recovery rates (Fig. 8 and Table S6, supplementary material) for Sketa 22, both SARS-CoV-2 genes and PMMoV reinforcing the need for mechanical homogenisation for environmental samples. It is important to recall that the MS2 internal control is added into the samples after the bead-beating step, therefore assessing the by-products after bead-beating rather than the impact of bead beating itself. The reduced Cq values for MS2 seen with mechanical homogenisation could be attributed to the other organisms, including inhibitors, or organic components abundant in wastewater being released in the lysis buffer more effectively during this process [19].

The series of tests conducted showed that the BoSL Beater 3D performed very similarly to the traditional FastPrep-24 bead beater (Fig. 8 and Table S6 in supplementary material). Neither the method of homogenisation nor the duration of the homogenisation step had a significant impact on the final Cq values of both SARS-CoV-2 genes; p = 0.10 and p = 0.27 for the different durations tested and p = 0.51 and p = 0.25 for homogenisation methods for SARS-CoV-2 N and ORF-1ab, respectively. On the other hand, the same parameters showed to significantly impact the Cq values for both PMMoV and Sketa 22 (p < 0.05 for both duration and instrument, with the BoSL Beater 3D recovering significantly higher rates, i.e. significantly lower Cq value).

**Fig. 9:**
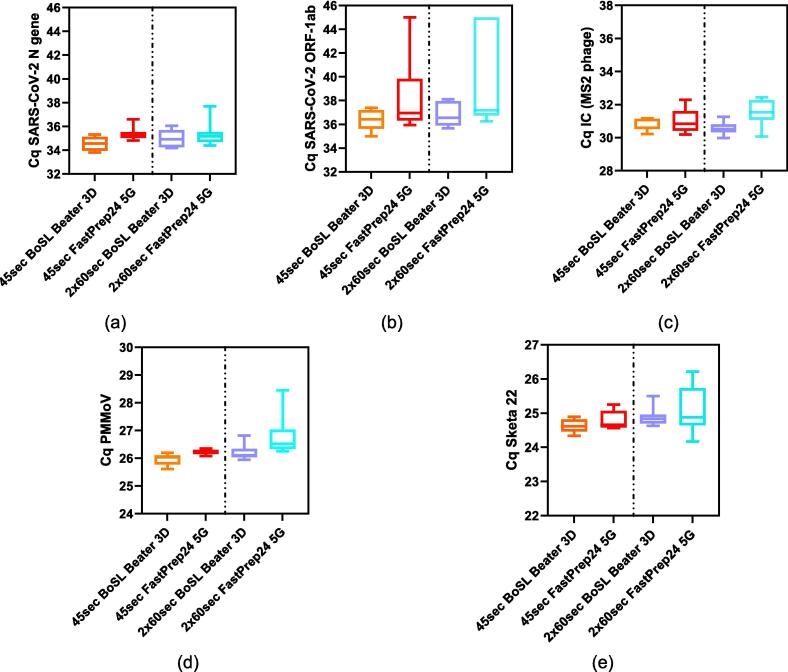
Combined Cq values (n = 5) obtained from the **0.45 µm passive samplers' CN membranes** for (a) SARS-CoV-2 N gene, (b) SARS-CoV-2 ORF-1ab gene, (c) Internal control – MS2 phage, (d) PMMoV and (e) Sketa22. Each boxplot represents the combined data for a given homogenisation method.

*Extraction efficiency and qPCR for passive samplers:* The combined data from all experiments is presented in boxplots in Fig. 9 and summary statistics are presented in Table S7 of the supplementary material. The results generated using the BoSL Beater 3D were similar or better than the traditional laboratory bead beater. Indeed, in all cases the mean Cq values from the BoSL Beater 3D samples were lower than those of the traditional laboratory instrument (Fig. 9 and Table S7 in supplementary material), suggesting a better recovery from the portable device. We identified significant differences in the mean Cq values between the four different homogenisation configurations for SARS-CoV-2 N gene (p = 0.04), PMMoV (p < 0.001) and MS2 (p = 0.04). In the case of MS2, the difference was driven by the comparison of the two instruments for a duration of 2 x 60 sec. As previously mentioned, it is important to note that the internal control was added into the samples after the bead-beating step, therefore assessing the by-products of bead-beating rather than the impact of bead beating itself and the difference could be explained by a higher concentration of inhibitors or organic compound being releases from the passive sampler material into the lysis buffer.

In comparison to the wastewater samples, we observed larger variation of the Cq values, which could be explained by the adsorption variability of the microbes onto the passive sampler material in comparison to how evenly distributed the microbes are when performing filtration. In addition, we observed larger Cq variation for the samples processed using the FastPrep-24 bead beater and lower variation for the BoSL Beater 3D, especially in the case of a longer homogenisation duration and for some of the targets (i.e., ORF-1ab, PMMoV, and Sketa22, Fig. 9b, d and e respectively). This reinforces the repeatability of the portable device developed in this paper for the extraction of passive samplers.

Overall, our BoSL Beater 3D performed similarly or better than the traditional benchtop bead beater for both DNA and RNA extraction in the context of wastewater surveillance. We have shown that the BoSL Beater 3D provides DNA and RNA extraction with good yield and quality leading to high-quality qPCR analysis. Our data indicates that the BoSL Beater 3D is a cost-effective, portable, adaptable, field-friendly alternative to a laboratory bead beating instrument. This tool in combination to newly developed field extraction kits could facilitate sample transport from remote areas or allow transfer from low-income countries to be tested in other facilities. With the addition of newly developed portable PCR instruments, this tool could be an asset for developing countries by allowing easier access to molecular-based techniques to process complex environmental samples as well as reduce reporting time and the risk of sample degradation in the context of remote areas without the need of sending large samples to equipped laboratories.

## 9 Capabilities and limitations of the hardware

*Longevity testing:* The lifespan of the BoSL Beater 3D was tested as if it was used in the laboratory with back-to-back repeated runs rather than continuous usage thus allowing us to assess the impact of stops and starts on each of the hardware’s parts. Each run was between 45 sec and 2 min, similarly to the most common requirement of the extraction kits used in our laboratory. After each run, the bead beater was visually inspected for wear and tear. The moment one of the components showed signs of deterioration, the total number of min of usage was calculated and gave us the lifespan of the 3D bead-beater attachment. On average, the jigsaw battery could perform for 15 min without showing signs of slowing down and was replaced to ensure we could maintain a consistent speed. It is important to note that these tests did not aim to assess the lifespan of the jigsaw itself and rather focused on the 3D printed parts only, especially the tube holder and lid. The test was performed on a total of three independent BoSL Beater 3D attachments.

This design was tested for a period of 4.6 h, which is equivalent to approximately 374 x 45 sec standard homogenization runs for extraction, or the extraction of over 2200 samples without showing signs of failure.

To the authors knowledge, other portable homogenizers exist and are either 25 times more expensive than the BoSL Beater 3D (comparison made with MPBio SuperFastPrep-2™), or have been discontinued due to safety reasons and battery limitations (only 20 min of operation for the Terralyser, Zymo, [20]). Other portable homogeniser have been developed but have only been tested for 200 min and have been developed to match a 20 min homogenization via vortexing [1] vs 45 sec to 2 min for the BoSL Beater 3D.

*Limitations: The limitations of the hardware include:*

• Speeds are dictated by the jigsaw utilised;
• Fits 1.5 and 2 mL extraction tubes only but another adapter could easily be designed and printed for larger tubes;
• Max 6 samples in 2 mL tubes can be processed at one time;
• Noise level of the jigsaw could be bothersome;
• Whilst more testing is still needed to assess the performances of the BoSL Beater 3D with other type of samples (such as other passive materials, soil and faeces), this study provides a proof of concept for a low-cost 3D printed bead beater for DNA and RNA extraction for a defined set of targets.
• The jigsaw blade can present a real hazard, a protective guard has been designed for this jigsaw model and would need to be adapted for other jigsaws.
• Only one design for one specific jigsaw currently exists, but our designs are easily available for modification to suit a range of jigsaws.

## 10 Ethics statements – Human and animal rights

Not Applicable. All samples collected and used in this study were collected from a local regional wastewater treatment plant which cannot be linking to any individual or animal.

## Credit authorship contribution statement

**Christelle Schang:** Writing – review & editing, Writing – original draft, Visualization, Validation, Project administration, Methodology, Investigation, Formal analysis, Data curation, Conceptualization. **Le Zhang:** Methodology, Conceptualization. **Baiqian Shi:** Writing – review & editing, Formal analysis, Conceptualization. **Monica Nolan:** Writing – review & editing. **Rachael Poon:** Writing – review & editing, Funding acquisition. **David T. McCarthy:** Writing – review & editing, Writing – original draft, Visualization, Validation, Supervision, Resources, Project administration, Methodology, Investigation, Formal analysis, Conceptualization.

## Declaration of competing interest

The authors declare that they have no known competing financial interests or personal relationships that could have appeared to influence the work reported in this paper.
