# Supplementary material for: A low-cost homogenizing device for in-field and remote DNA and RNA extraction
Source: HardwareX. 2024 Oct 9;20:e00592. doi: 10.1016/j.ohx.2024.e00592 (PMC11532470; doi:10.1016/j.ohx.2024.e00592)
Supplement: Supplementary Data 1 [file mmc1.docx]

Supplementary material

**Manuscript title:** A low-cost homogenizing device for in-field and remote DNA and RNA extraction

**Authors:** Christelle Schang^1^, Le Zhang^1^, Baiqian Shi^1^, Monica Nolan^1^, Rachael Poon^2^, David T. McCarthy^1,3, 4^*

1. *BoSL Water Monitoring and Control, Department of Civil Engineering, Monash University, Victoria 3800, Australia*
2. *Department of Health Victoria, Melbourne, Victoria, 3000, Australia.*
3. *School of Civil and Environmental Engineering, QUT, Brisbane, QLD 4001, Australia*
4. *School of Environmental Sciences, Ontario Agricultural College, University of Guelph, Canada*

**Corresponding author. E-mail address: david.mccarthy@uoguelph.ca* *(D.T. McCarthy).*

Number of pages: 6

Number of figures: 2

Number of tables: 7

**Hardware description**

| 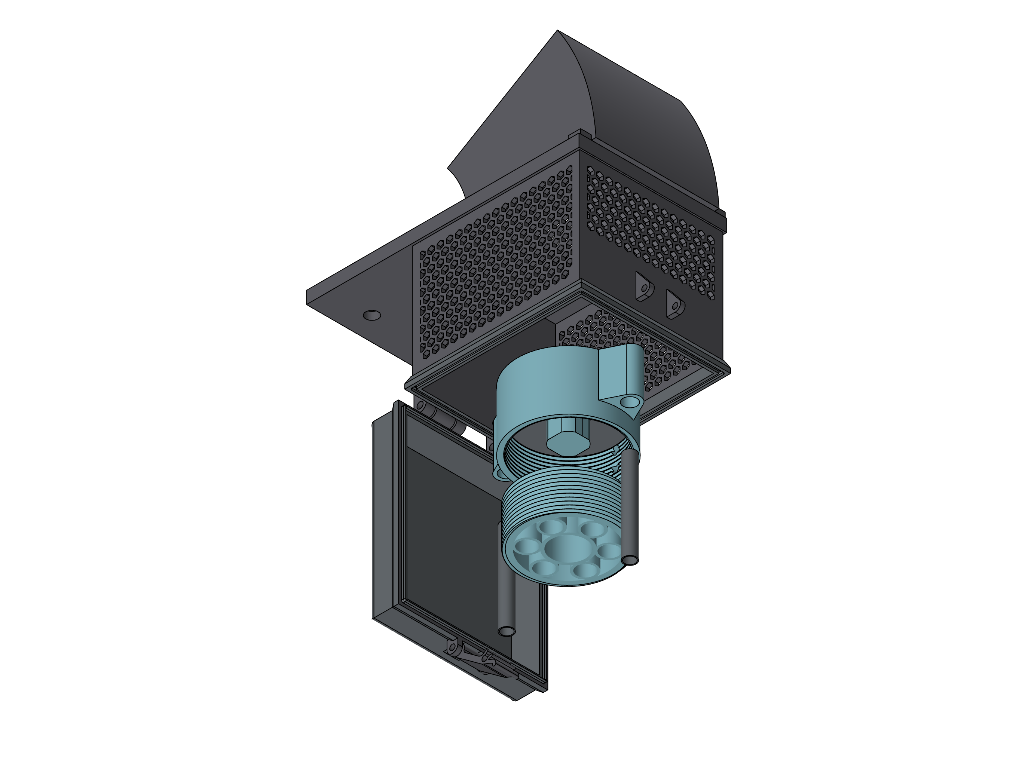 | 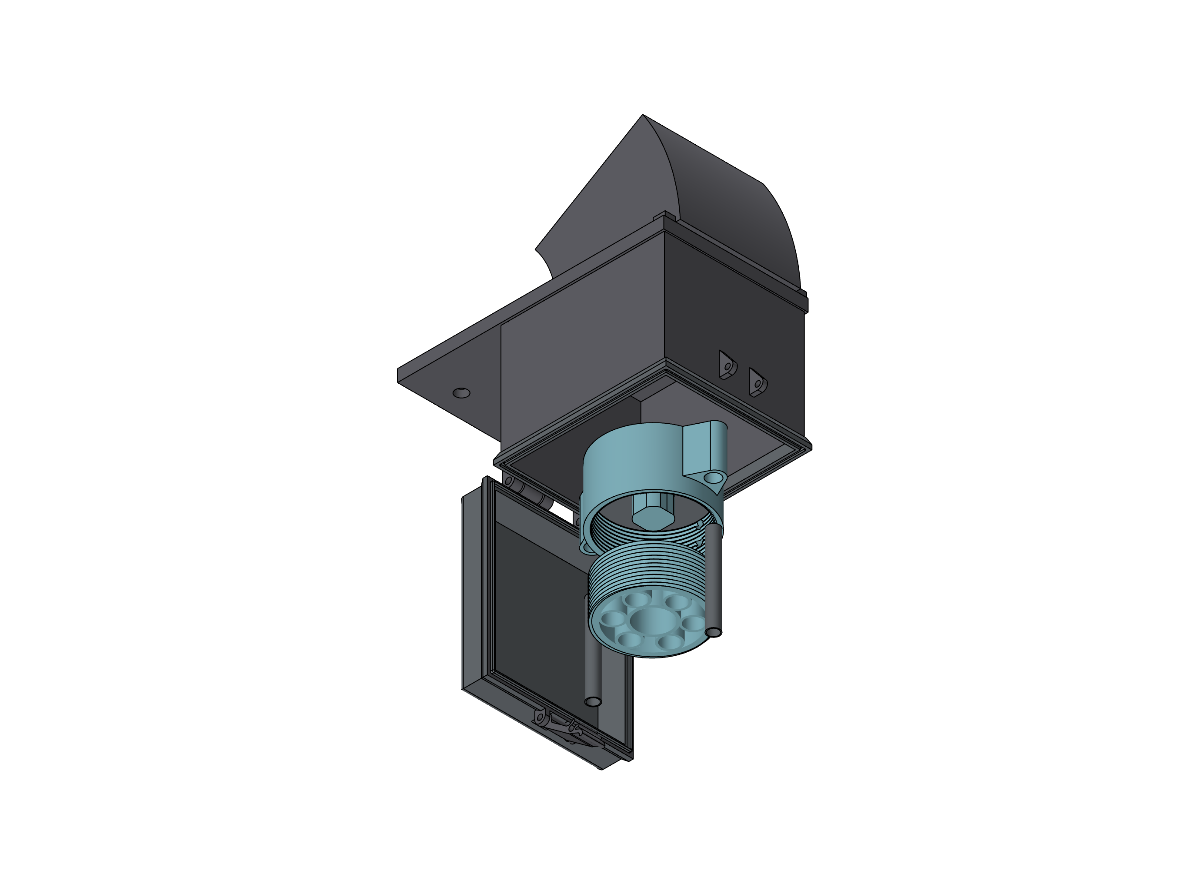 |
| --- | --- |

Figure S1. 3D rendering of the BoSL Beater 3D with the honeycomb protective case (left) and solid wall protective case (right).

**Real-Time quantitative PCR.**

Quantitative real-time PCR (qPCR) used for the detection and quantification of SARS-CoV-2 using the Perkin Elmer SARS-CoV-2 Real Time RT-PCR assay (PerkinElmer, Shelton, USA). This assay targets the nucleocapsid (hereby N) and ORF-1ab genomic regions of the SARS-CoV-2 virus. This assay used MS2 bacteriophage as an internal process control (IC) that is added in a known amount to each of the samples after the bead beating process as described in [1]. As described in Schang et al. [1] and Gazeley et al. [2], the volume of reagents used in each qPCR reaction was halved in comparison to the volume recommended by the manufacturer. Sequences of the primers and probe for the two SARS-CoV-2 genes of interest in this commercial kit, identified as the China CDC set by Water Research Australia [3] and described in Suo et al. [4], are summarised in Table S1. Due to the proprietary nature of the Perkin Elmer kit, the concentration and probe quenchers of all primers and probes and chemistry of the reagents as well as the detailed sequences for the IC control and its targeting primers are not disclosed. Sequences of the primers and probes for PMMoV taken from Hsu et al. [5] and the sample processing control sequence (SPC) taken from chum salmon, *Oncoryhynchus keta* from the US EPA Method 1696 [6] are also summarised in Table S1.

Table S1. Primers and probes Oligonucleotide sequences for SARS-CoV-2 nucleocapsid (N) and ORF-1ab targets within the Perkin Elmer SARS-CoV-2 Real-time RT-PCR assay as determined by Water Research Australia [3] and described by Suo at al., 2020 [4] and pepper mild motile virus (PmmoV) as determined by Hsu et al., 2022[5].

| **Target** | **Sequence (5’–3’)** | **Primer/probe concentration**  **(uM)** | **Amplicon length**  **(bp)** | **Reference** |
| --- | --- | --- | --- | --- |
| SARS-CoV-2 – Nucleocapside (N) | F: GGGGAACTTCTCCTGCTAGAAT  R: CAGACATTTTGCTCTCAAGCTG  P: [FAM]-TTGCTGCTGCTTGACAGATT | Unknown | Unknown | Perkin Elmer |
| SARS-CoV-2  ORF-1ab | F: CCCTGTGGGTTTTACACTTAA  R: ACGATTGTGCATCAGCTGA  P: [ROX]-CCGTCTGCGGTATGTGGAAAGGTTATGG | Unknown | Unknown | Perkin Elmer |
| MS2 phage | Unknown | Unknown | Unknown | Perkin Elmer |
| PMMoV | F: GGCGTAGATCCATTGGTGG | 250nM | 180 | [5] |
|  | R: CGAACCTTCCTCCTTTGATG | 250nM |  |  |
|  | P: Cy5-5’ GCTGTGGTTTCAAATGAGAGTGG 3’-QSY | 62.5nM |  |  |
|  | R: TTCCTCTCAGAACCCCTATCC | 1µM |  |  |
|  | P: [6-FAM]-5'-CTAATGGAACGCATCCC-MGB | 80nM |  |  |

The details of the reagents used in our qPCR protocols are provided in Table S2. The parameters used for our thermocycling and qPCR assays are presented in Figure S1.

Table S2. RT-qPCR mastermix used in this study.

| **Reagent name** | **Volume per reaction** |
| --- | --- |
| nCoV Reagent A | 7.5 μL |
| nCoV Reagent B | 1.5 μL |
| nCoV Enzyme Mix | 1 μL |
| PMMoV primer and probe mix | 0.75 µL |
| UltraPure DNase/RNase free water | 15 μL |
| RNA template | 5 μL |

Figure S2. SARS-CoV-2 and PMMoV RT qPCR Run for amplification and fluorescence detection on BIORAD CFX 96 (based on Instructions for Perkin Elmer®SARS-CoV-2 Real-time RT-PCR Assay, reaction volume 30 μL). Image taken from Bio-Rad CFX Maestro software version 2.3 (5.3.022.1030)


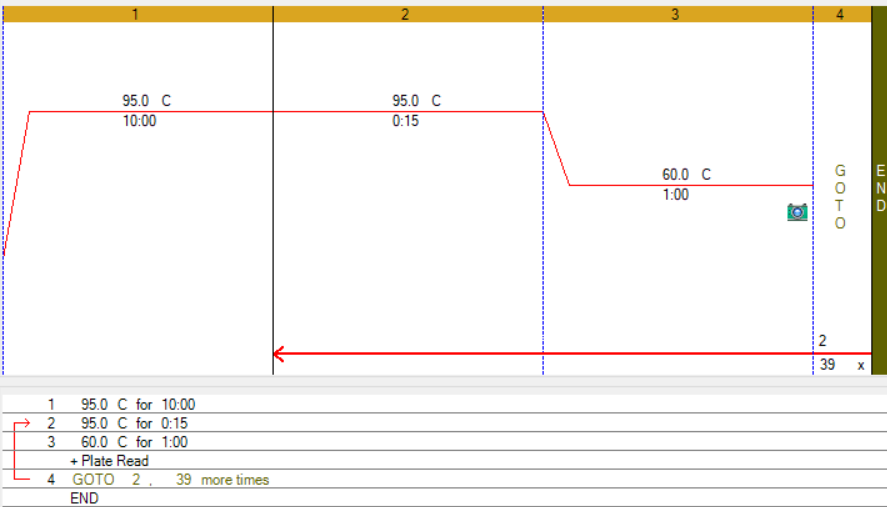


Figure S3.Sketa22 qPCR Run for amplification and fluorescence detection on BIORAD CFX 96 (based on Instructions from USEPA Method 1696 [6], reaction volume 25 μL). Image taken from Bio-Rad CFX Maestro software version 2.3 (5.3.022.1030)

**Statistical analysis**

Statistical analysis was performed using GraphPad Prism (version 9.0.1 (151), GraphPad Software, LLC) and Microsoft Excel (Version 2407, Microsoft 365 MSO) to assess the impact of the homogenisation methods, durations of the homogenisation and sample types on the DNA and RNA quality and the Cq values for the different organisms of concerns. The data set had to be fractioned as the experimental design was not full-factorial (Table S3).

Table S3.Summary of all factors tested in the laboratory testing and validation of the 3D BoSl-Beater. The variable name is provided together with the number of possible variations in brackets and the variation are detailed in the second column.

| **Variable** | **Non-Factorial factors** |
| --- | --- |
| Homogenisation methods | FastPrep-24™ 5G bead beating grinder and lysis system, BoSL-beater-3D, manual shaking |
| Homogenisation duration | 10 sec, 45 sec, 60 sec, 2x60 sec with 1min rest |
| Sample type | 20 mL wastewater, CN membrane passive sample |
| qPCR Target type | SARS-CoV-2 N gene, SARS-CoV-2 ORF-1ab gene, MS2 Phage (IC), Sketa22, PMMoV |

**DNA and RNA quality**

*DNA quality:* Summary statistics of the DNA quality measurements for both the wastewater samples and the passive samples are provided Table S4. In general, an A_260_/A_280_ ratio of ~1.8 is generally accepted as “pure” for DNA [7]. Overall, the use of the BoSL-Beater 3D resulted in samples of higher purity than those obtained from the FastPrep-24 5G instrument for the wastewater samples, whilst the reverse was observed for the passive samplers. With DNA A_260_/A_280_ ratios ranging from 1.90 to 1.99, our wastewater samples were considered as pure for all bead beating conditions and instrument, whilst the passive sampler the DNA A_260_/A_280_ ratios were just below 1.9 for the samples processed using the BoSL Beater 3D for both durations tested. The A_260_/A_230_ ratio for the wastewater samplers ranged from 1.98 to 2.10 showing little to no organic contamination in these samples. On the other hand, passive samplers had A_260_/A_230_ ratios ranging from 1.13 to 1.62 indicating the potential presence of unwanted organic compounds and chaotropic salt in the passive samplers’ extracts. This could be due to the method of extraction and the nature of the samples rather than the homogenisation method, which could have resulted in more residual guanidine from the lysis buffer for the passive samples than the wastewater samples [7]. Overall, there were less traces of organic compounds in the extracts obtained using the BoSL-Beater 3D for the homegenisation step which could be explained from the lower speed resulting in less shearing of the grinding material which has been reported to possibly cause contamination [8].

*Table S4. DNA extraction quality comparison. Descriptive statistic for DNA yield, purity ratio A_260_/A_280_ and organic contamination assessment ratio A_260_/A_230_ for the 20mL wastewater (n=9 samples) and passive samplers (n=5 samples) extracted using different homogenisation methods. “-“ indicates that this setting was not tested*

|  |  |  |  | **MPBio FastPrep24 5G** | | | **BoSL-Beater-3D** | | | **10 manual shakes** | | | |
| --- | --- | --- | --- | --- | --- | --- | --- | --- | --- | --- | --- | --- | --- |
| **Sample type** | **Homogenisation method** | N |  | DNA Yield | DNA  A_260_/A_280_ | DNAA_260_/A_230_ | DNA Yield | DNA  A_260_/A_280_ | DNAA_260_/A_230_ | N | DNA Yield | DNA  A_260_/A_280_ | DNAA_260_/A_230_ |
|  |  |  |  | (ng/uL) |  |  | (ng/uL) |  |  |  | (ng/uL) |  |  |
| 20mL wastewater | 10sec bead beating | 9 | Av | 197.96 | 1.99 | 2.10 | 261.97 | 2.00 | 2.10 | 3 | 86.86 | 1.95 | 2.16 |
|  |  |  | St dev | 85.26 | 0.03 | 0.17 | 35.66 | 0.03 | 0.12 |  | 23.96 | 0.02 | 0.16 |
|  | 45sec bead beating | 9 | Av | 247.85 | 1.96 | 2.01 | 405.61 | 1.98 | 1.98 | - | - | - | - |
|  |  |  | St dev | 38.29 | 0.04 | 0.19 | 170.45 | 0.01 | 0.19 | - | - | - | - |
|  | 60sec bead beating | 9 | Av | 285.98 | 1.96 | 1.99 | 413.13 | 1.98 | 2.02 | - | - | - | - |
|  |  |  | St dev | 79.38 | 0.01 | 0.13 | 103.63 | 0.02 | 0.11 | - | - | - | - |
| Passive samplers | 45sec bead beating | 5 | Av | 7.35 | 1.93 | 1.13 | 7.59 | 1.88 | 1.62 | - | - | - | - |
|  |  |  | St dev | 2.87 | 0.26 | 0.56 | 0.62 | 0.16 | 0.28 | - | - | - | - |
|  | 2x60sec bead beating | 5 | Av | 7.12 | 1.90 | 1.39 | 6.65 | 1.86 | 1.53 | - | - | - | - |
|  |  |  | St dev | 3.07 | 0.39 | 0.35 | 1.35 | 0.15 | 0.29 | - | - | - | - |

*RNA quality:* Summary statistics of the RNA quality measurements for both the wastewater samples and the passive samples are provided Table S5. In terms of quality, a A_260_/A_280_ of ~2.0 is generally accepted as “pure” for RNA [7]. The wastewater samples RNA extracts all had a ratio close to 2.0 and showed that although, not statistically significant, the duration of the bead beating step was the most important parameter impacting purity of the samples with the 60 sec bead beating resulting in a lower ratio than for 10 sec. The RNA quality of the extracts obtained from passive samplers all had a significantly lower ratio than those obtained from wastewater samples (p<0.005), on average the A_260_/A_280_ RNA ratios were 1.45 for the FastPrep24 5G and 1.75 for the BoSL-Beater 3D showing that the samples processed using the BoSL Beater 3D had a higher purity than those processed with the FastPrep-24. The lower A_260_/A_280_ for passive samples, can be explained by the lower amount of material that has accumulated onto the CN membranes in comparison the 20mL of wastewater directly filtered onto the membranes or by more contamination due to wear of the grinding media [8] as the garnet beads can move more freely within the tubes containing the passive sampler membrane.

*Table S5. RNA extraction quality comparison. Descriptive statistic for RNA yield, purity ratio A_260_/A_280_ and organic contamination assessment ratio A_260_/A_230_ for the 20mL wastewater (n=9 samples) and passive samplers (n=5 samples) extracted using different homogenisation methods. “-“ indicates that this setting was not tested*

|  |  |  |  | **MPBio FastPrep24 5G** | | | **BoSL-Beater-3D** | | | **10 manual shakes** | | | |
| --- | --- | --- | --- | --- | --- | --- | --- | --- | --- | --- | --- | --- | --- |
| **Sample type** | **Homogenisation method** | N |  | RNA Yield | RNA  A_260_/A_280_ | RNA  A_260_/A_230_ | RNA Yield | RNA  A_260_/A_280_ | RNA  A_260_/A_230_ | N | RNA Yield | RNA  A_260_/A_280_ | RNA  A_260_/A_230_ |
|  |  |  |  | (ng/uL) |  |  | (ng/uL) |  |  |  | (ng/uL) |  |  |
| 20mL wastewater | 10sec bead beating | 9 | Av | 163.11 | 1.96 | 1.98 | 209.05 | 1.99 | 2.00 | 3 | 70.88 | 1.96 | 1.98 |
|  |  |  | St dev | 77.53 | 0.05 | 0.04 | 24.79 | 0.03 | 0.03 |  | 20.08 | 0.02 | 0.02 |
|  | 45sec bead beating | 9 | Av | 198.55 | 1.95 | 1.97 | 324.10 | 1.98 | 1.99 | - | - | - | - |
|  |  |  | St dev | 31.32 | 0.02 | 0.02 | 138.76 | 0.02 | 0.02 | - | - | - | - |
|  | 60sec bead beating | 9 | Av | 233.89 | 1.93 | 1.95 | 332.99 | 1.97 | 1.99 | - | - | - | - |
|  |  |  | St dev | 65.87 | 0.02 | 0.02 | 85.52 | 0.04 | 0.03 | - | - | - | - |
| Passive samplers | 45sec bead beating | 5 | Av | 6.12 | 1.56 | 1.68 | 5.66 | 1.69 | 1.96 | - | - | - | - |
|  |  |  | St dev | 1.93 | 0.23 | 0.25 | 0.38 | 0.14 | 0.19 | - | - | - | - |
|  | 2x60sec bead beating | 5 | Av | 5.34 | 1.34 | 1.49 | 4.79 | 1.80 | 2.24 | - | - | - | - |
|  |  |  | St dev | 2.54 | 0.12 | 0.17 | 0.96 | 0.09 | 0.32 | - | - | - | - |

**Extraction efficiency and qPCR for filtered wastewater samples and passive samples**

Table S6. Summary statistics Cq values for the five qPCR target tested on the 20ml wastewater samples filtered on CN membranes. The data from 4 tests is included into the analysis. Bold and underline information indicates when the geomean Cq values were better for the BoSL Beater 3D in comparison to the laboratory instrument.

|  |  |  |  | **Cq value 20mL wastewater on CN membrane** | | | |
| --- | --- | --- | --- | --- | --- | --- | --- |
| **Target** | **Homogenisation duration** | **Homogenisation method** | **n** | **Geomean** | **Geometric SD factor** | **Min** | **Max** |
| SARS-CoV-2 N | 10 shakes | Manual | 6 | 32.7 | 1.06 | 30.4 | 34.3 |
|  | 10 sec | BoSL Beater 3D | 18 | 32.3 | 1.04 | 30.4 | 34.3 |
|  |  | FastPrep24 | 18 | 32.1 | 1.05 | 29.3 | 35.2 |
|  | 45 sec | BoSL Beater 3D | 18 | 32.9 | 1.04 | 30.5 | 36.4 |
|  |  | FastPrep24 | 18 | 32.2 | 1.03 | 30.1 | 33.2 |
|  | 60 sec | BoSL Beater 3D | 18 | **32.8** | 1.04 | 29.5 | 35.2 |
|  |  | FastPrep24 | 18 | 33.1 | 1.04 | 31.2 | 34.9 |
| SARS-CoV-2 ORF-1ab | 10 shakes | Manual | 6 | 34.4 | 1.04 | 32.8 | 35.7 |
|  | 10 sec | BoSL Beater 3D | 18 | **33.1** | 1.04 | 30.5 | 35.7 |
|  |  | FastPrep24 | 18 | 33.4 | 1.03 | 31.4 | 35.1 |
|  | 45 sec | BoSL Beater 3D | 18 | 33.6 | 1.05 | 31.3 | 37.1 |
|  |  | FastPrep24 | 18 | 33.1 | 1.03 | 30.7 | 34.7 |
|  | 60 sec | BoSL Beater 3D | 18 | **33.1** | 1.06 | 29 | 36.7 |
|  |  | FastPrep24 | 18 | 33.5 | 1.03 | 31.3 | 35.5 |
| IAC (MS2 phage) | 10 shakes | Manual | 6 | 29.5 | 1.02 | 29.1 | 30.2 |
|  | 10 sec | BoSL Beater 3D | 18 | 30.2 | 1.04 | 27.9 | 32.2 |
|  |  | FastPrep24 | 18 | 29.9 | 1.05 | 28.9 | 33.8 |
|  | 45 sec | BoSL Beater 3D | 18 | 30.8 | 1.06 | 28.8 | 35.5 |
|  |  | FastPrep24 | 18 | 30.3 | 1.03 | 29.1 | 32.3 |
|  | 60 sec | BoSL Beater 3D | 18 | **30.9** | 1.02 | 29.7 | 31.9 |
|  |  | FastPrep24 | 18 | 31.2 | 1.05 | 29.4 | 34.8 |
| PMMoV | 10 shakes | Manual | 6 | 24.2 | 1.01 | 23.9 | 24.6 |
|  | 10 sec | BoSL Beater 3D | 18 | **23.1** | 1.02 | 22.5 | 24.4 |
|  |  | FastPrep24 | 18 | 23.4 | 1.02 | 22.8 | 25.1 |
|  | 45 sec | BoSL Beater 3D | 18 | 23.7 | 1.04 | 22.7 | 25.4 |
|  |  | FastPrep24 | 18 | 23.5 | 1.02 | 23 | 24.3 |
|  | 60 sec | BoSL Beater 3D | 18 | **23.5** | 1.04 | 22.7 | 26.3 |
|  |  | FastPrep24 | 18 | 23.5 | 1.01 | 23.2 | 24.3 |
| Sketa 22 | 10 shakes | Manual | 6 | 23.5 | 1.01 | 23.3 | 23.6 |
|  | 10 sec | BoSL Beater 3D | 18 | **23.4** | 1.02 | 22.8 | 24.5 |
|  |  | FastPrep24 | 18 | 23.4 | 1.02 | 22.8 | 24.3 |
|  | 45 sec | BoSL Beater 3D | 18 | **23.2** | 1.01 | 22.6 | 24 |
|  |  | FastPrep24 | 18 | 23.3 | 1.01 | 22.7 | 23.7 |
|  | 60 sec | BoSL Beater 3D | 18 | **23.2** | 1.03 | 22.6 | 25.2 |
|  |  | FastPrep24 | 18 | 23.2 | 1.01 | 22.7 | 23.8 |

Table S7. Summary statistics Cq values for the five qPCR targets tested on the passive CN membranes exposed to wastewater. The data from 4 tests is included into the analysis. Bold and underline information indicates when the geomean Cq values were better for the BoSL Beater 3D in comparison to the laboratory instrument.

|  |  |  |  | **Cq value Passive CN membranes** | | | |
| --- | --- | --- | --- | --- | --- | --- | --- |
| **Target** | **Homogenisation duration** | **Homogenisation method** | **n** | **Geomean** | **Geometric SD factor** | **Min** | **Max** |
| SARS-CoV-2 N | 45 sec | BoSL Beater 3D | 10 | **34.5** | 1.02 | 33.8 | 35.3 |
|  |  | FastPrep24 | 10 | 35.3 | 1.01 | 34.8 | 36.6 |
|  | 2x60 sec | BoSL Beater 3D | 10 | **35** | 1.02 | 34.2 | 36 |
|  |  | FastPrep24 | 10 | 35.3 | 1.03 | 34.4 | 37.7 |
| SARS-CoV-2 ORF-1ab | 45 sec | BoSL Beater 3D | 10 | **36.4** | 1.02 | 35 | 37.4 |
|  |  | FastPrep24 | 10 | 38.4 | 1.09 | 35.9 | 45 |
|  | 2x60 sec | BoSL Beater 3D | 10 | **36.8** | 1.03 | 35.7 | 38.1 |
|  |  | FastPrep24 | 10 | 39.2 | 1.1 | 36.3 | 45 |
| IAC (MS2 phage) | 45 sec | BoSL Beater 3D | 10 | **30.9** | 1.01 | 30.2 | 31.2 |
|  |  | FastPrep24 | 10 | 31 | 1.02 | 30.2 | 32.3 |
|  | 2x60 sec | BoSL Beater 3D | 10 | **30.6** | 1.01 | 30 | 31.3 |
|  |  | FastPrep24 | 10 | 31.5 | 1.03 | 30.1 | 32.4 |
| PMMoV | 45 sec | BoSL Beater 3D | 10 | **26.2** | 1.01 | 25.9 | 26.5 |
|  |  | FastPrep24 | 10 | 26.5 | 1 | 26.4 | 26.7 |
|  | 2x60 sec | BoSL Beater 3D | 10 | **26.5** | 1.01 | 26.2 | 27.1 |
|  |  | FastPrep24 | 10 | 27.1 | 1.03 | 26.5 | 28.8 |
| Sketa 22 | 45 sec | BoSL Beater 3D | 10 | **24.6** | 1.01 | 24.3 | 24.9 |
|  |  | FastPrep24 | 10 | 24.8 | 1.01 | 24.6 | 25.3 |
|  | 2x60 sec | BoSL Beater 3D | 10 | **24.9** | 1.01 | 24.6 | 25.5 |
|  |  | FastPrep24 | 10 | 25.1 | 1.03 | 24.2 | 26.2 |

**References**

1. Schang, C., et al., *Passive sampling of SARS-CoV-2 for wastewater surveillance.* Environmental science & technology, 2021. **55**(15): p. 10432-10441.

2. Gazeley, J., et al., *Correlating Quantitative and Genomic SARS-CoV-2 Wastewater Data with Clinical Metrics in Metropolitan Perth, Western Australia.* Environments, 2024. **11**(4): p. 62.

3. Water Research Australia, *ColoSSoS|Method Evaluation and Optimisation: Investigation of PCR-Based Methods and Feasibility Study for Whole-Genome Sequencing.* 2021.

4. Suo, T., et al., *ddPCR: a more accurate tool for SARS-CoV-2 detection in low viral load specimens.* Emerging microbes & infections, 2020. **9**(1): p. 1259-1268.

5. Hsu, S.-Y., et al., *Biomarkers selection for population normalization in SARS-CoV-2 wastewater-based epidemiology.* Water research, 2022. **223**: p. 118985.

6. USEPA, *Method 1696: Characterization of Human Fecal Pollution in Water by HF183/BacR287 TaqMan® Quantitative Polymerase Chain Reaction (qPCR) Assay*. 2019.

7. Matlock, B., *Assessment of nucleic acid purity.* Technical Note, 2015. **52646**: p. 1-2.

8. Goldberg, S., *Mechanical/physical methods of cell disruption and tissue homogenization.* 2D PAGE: Sample preparation and fractionation, 2008: p. 3-22.
